# The West London Hospital

**Published:** 1916-11-25

**Authors:** Percy Dunn

**Affiliations:** Wimpole Street, W.


### The West London Hospital.

*To the Editor of* The Hospital.

Sir,—Perhaps you will permit me to make a few observations in reply to your friendly remarks* upon my last instalment "In Earlier Days," contributed to the *West London Medical Journal*? The burden of my commentary in reference to the controlling influence of a chairman over a hospital board was not to show that "the tendency of the board at the West London was to override the recommendations of the medical committee." That would have been to create an entirely contrary impression. I sought, however, to submit that the action of any hospital board in this regard could not be held to be venial. Taking a broad-minded view of the facts, the following reasons subtend the situation. First, there is in the case of all hospital boards an inner official ring, which, like the board of directors of a company, considers and determines the policy of management of the institution concerned. In all such "rings" there must be a leader, and naturally that position commonly falls to the chairman of the hospital, whose personal activities in promoting the success of the institution may have earned for him the approval, the loyal recognition, and appreciation of his colleagues. Naturally, therefore, it is inconceivable that any such chairman, with the power he had acquired, would be likely to imperil the good interests of his hospital by exercising that power unworthily. Moreover, when the difference between highly recommended candidates for a vacancy practically amounts to the difference between Tweedledum and Tweedledee, as is commonly the case, the question may be asked, Can a chairman be held to have acted venially by securing the election of one candidate at the expense of the rest? There is nothing which wounds more deeply a man's *amour propre* than to fail for an appointment, and nothing which deters him from seeking a scapegoat to explain his discomfiture. I am, however, in complete agreement with your remark that this subject "is somewhat a delicate matter." Nevertheless, the fact remains that where a hospital chairman has gained the reputation of controlling the suffrages of his board, ample experience has shown that the ascendancy he has acquired has been due to his devoted personal services to the institution.

In conclusion, I desire again to repeat that, judging from an experience of thirty years, the West London Hospital has been admirably administered in the past, and is so still, by its house committee and board of management.—I am, Sir, yours truly, Percy Dunn.

Wimpole Street, W., November 21, 1916.

[We thank Mr. Dunn for his letter, which seems to us a most judicious pronouncement. We are pleased to find this acute observer so nearly in agreement with us on the topic under discussion.—Ed. The Hospital.]

* The Hospital, November 11, p. 112.

### Sanatorium Buildings at £12 per Bed.

*To the Editor of* The Hospital.

